# Pancreatic cancer triggers diabetes through TGF-β–mediated selective depletion of islet β-cells

**DOI:** 10.26508/lsa.201900573

**Published:** 2020-05-05

**Authors:** Parash Parajuli, Thien Ly Nguyen, Céline Prunier, Mohammed S Razzaque, Keli Xu, Azeddine Atfi

**Affiliations:** 1 Cellular and Molecular Pathogenesis Division, Department of Pathology and Massey Cancer Center, Virginia Commonwealth University, Richmond, VA, USA; 2 Cancer Institute, University of Mississippi Medical Center, Jackson, MS, USA; 3 Sorbonne Université, Inserm, Centre de Recherche Saint-Antoine, Paris, France; 4 Department of Pathology, Lake Erie College of Osteopathic Medicine, Erie, PA, USA

## Abstract

Pancreatic ductal adenocarcinoma (PDAC) is a lethal disease that remains incurable because of late diagnosis, which renders any therapeutic intervention challenging. Most PDAC patients develop de novo diabetes, which exacerbates their morbidity and mortality. How PDAC triggers diabetes is still unfolding. Using a mouse model of Kras^G12D^-driven PDAC, which faithfully recapitulates the progression of the human disease, we observed a massive and selective depletion of β-cells, occurring very early at the stages of preneoplastic lesions. Mechanistically, we found that increased TGF beta (TGF-β) signaling during PDAC progression caused erosion of β-cell mass through apoptosis. Suppressing TGF-β signaling, either pharmacologically through TGF-β immunoneutralization or genetically through deletion of *Smad4* or *TGF-*β *type II receptor* (*TβRII*), afforded substantial protection against PDAC-driven β-cell depletion. From a translational perspective, both activation of TGF-β signaling and depletion of β-cells frequently occur in human PDAC, providing a mechanistic explanation for the pathogenesis of diabetes in PDAC patients, and further implicating new-onset diabetes as a potential early prognostic marker for PDAC.

## Abstract (precis)

Pancreatic ductal adenocarcinoma formation culminates in hyperactivation of transforming growth factor β signaling, which in turn induces selective depletion of β-cell mass and thereby leading to new-onset diabetes.

## Introduction

Pancreatic ductal adenocarcinoma (PDAC) is one of the most lethal human malignancies, with a median survival of less than 6 mo and a cumulative 5-yr survival rate of 3–7% (Hidalgo, 2010). PDAC can progress from pancreatic intraepithelial neoplasia (PanIN), intraductal papillary mucinous neoplasia, or mucinous cystic neoplasia, although PanINs represent the most common precursor lesions (Hruban et al, 2004). The vast majority of PDAC tumors (∼90%) harbor activating mutations in the proto-oncogene *KRAS*, most notably KRAS^G12D^ (Almoguera et al, 1988; Hezel et al, 2006). Constitutively activated KRAS induces cancer cell proliferation by increasing glucose consumption approximately ten times more than in normal cells, a phenomenon known as the Warburg effect (Hsu & Sabatini, 2008; Ying et al, 2012). Given this dependency on glucose, one would surmise that hyperglycemia, which is typically associated with diabetes, could play an instrumental role in the pathogenesis and progression of PDAC by providing the glucose needed to fuel the growth of cancer cells. Interestingly, about 80% of PDAC patients develop type 2 diabetes (T2D), and PDAC incidence is two times higher in diabetic patients than in nondiabetic individuals (McAuliffe & Christein, 2013; Tan et al, 2017). Conversely, patients who are recently diagnosed with diabetes have 50% greater risk of developing PDAC than healthy individuals (Huxley et al, 2005; McAuliffe & Christein, 2013). Genetic studies using the *KPC* mouse model of PDAC showed that PDAC development did not exacerbate diabetes induced by high-fat diet (HFD) (Pasquale et al, 2019), hinting at the possibility that PDAC might induce diabetes without causing insulin resistance. Yet, the mechanisms underlying the detrimental association between PDAC and diabetes remain poorly understood.

Diabetes is a debilitating metabolic disease characterized by high blood glucose resulting from defects in insulin production, insulin signaling, or both. There are two broad etiopathogenetic categories of diabetes: type 1 diabetes (T1D), which results from absolute insulin deficiency, and T2D, which is caused by a combination of insulin resistance and inadequate insulin secreting compensation. T1D accounts for 5–10%, whereas T2D accounts for 90–95% of all diabetic patients (Ashcroft & Rorsman, 2012). The islets of Langerhans represent the endocrine system of the pancreas that plays a key role in the pathogenesis of both T1D and T2D. The islets of Langerhans consist mainly of α, β, δ, and pancreatic polypeptide (PP) cells, which produce glucagon, insulin, somatostatin, and PP, respectively (Bastidas-Ponce et al, 2017). Although these endocrine cells fulfill distinct functions, the interactions among them are crucial for maintaining whole-body glucose homeostasis (Jain & Lammert, 2009). For instance, insulin secreted by β-cells is responsible for the suppression of gluconeogenesis in the liver, whereas glucagon secreted by α-cells exerts the opposite effect. Currently, whether acquisition of oncogenic *KRAS* in the pancreatic epithelium affects the fate or function of any of those islet cells remains to be established.

Besides oncogenic mutations in *KRAS*, other genetic alterations that are deemed essential for the progression from early neoplastic lesions to invasive PDAC include loss-of-function mutations in the tumor suppressor *SMAD4* (also known as *DPC4*) and *TGF*-β *type II receptor* (*TβRII*), two essential components of the TGF-β signaling pathway (Iacobuzio-Donahue, 2012; Cancer Genome Atlas Research Network, 2017). TGF-β signaling regulates a wide variety of functions, including specification of cell fate during embryogenesis, proliferation, differentiation, and apoptotic cell death (Whitman, 1998; Feng & Derynck, 2005). TGF-β signaling pathway is initiated when the ligand induces assembly of a heteromeric TβRII and TβRI (TGF-β type I receptor) complex, thereby allowing phosphorylation and activation of the TβRI kinase by the constitutively active kinase of TβRII (Massague et al, 2005). The activated TβRI then propagates the signal to the nucleus by phosphorylating Smad2 and Smad3 on two serine residues (S465/S467) at their C termini. Once phosphorylated, Smad2 and Smad3 associate with the common partner Smad4, and the resulting complexes then translocate to the nucleus, where they regulate the expression of TGF-β target genes through cooperative interactions with transcriptional coactivators or corepressors (Massague et al, 2005). The interest in exploiting the TGF-β signaling pathway in cancer stems from its dichotomous role as a tumor suppressor and tumor promoter (Derynck et al, 2001; Massague, 2008). In fact, TGF-β suppresses proliferation or induces apoptosis in most normal epithelial cells, thus inhibiting tumor initiation. On the other hand, TGF-β can exacerbate the progression of already established tumors, thus acting as a pro-metastatic factor. Currently, the mechanistic underpinnings of this bimodal action of TGF-β signaling remain poorly understood.

During PDAC progression, TGF-β signaling becomes hyperactive because of increased TGF-β secretion from both cancer cells and their surrounding stroma, and this activation is known to be associated with poor survival in PDAC patients (Friess et al, 1993; Bardeesy et al, 2006). Of particular relevance, increased TGF-β signaling has been shown to occur in early PanINs in the absence of any apparent metastasis (Friess et al, 1993; Bardeesy et al, 2006), raising the possibility that activation of this pathway might also impinge on other vital physiological processes beyond simply suppressing cell proliferation and/or fostering cell invasion and metastasis. In this study, we combined several orthogonal approaches and genetically engineered mouse models to show that TGF-β signaling plays a causal role in the development of diabetes during PDAC progression. Most notably, we found that genetic inactivation of *Smad4* or *TβRII* in the Kras^G12D^ mouse model of human PDAC was sufficient to suppress PDAC-mediated diabetes. Likewise, immunoneutralization of TGF-β in vivo almost completely blunted PDAC-mediated diabetes, implicating TGF-β signaling as a possible target for attenuating diabetes in pancreatic cancer patients.

## Results

### PDAC affects islet integrity

**Figure S1.**
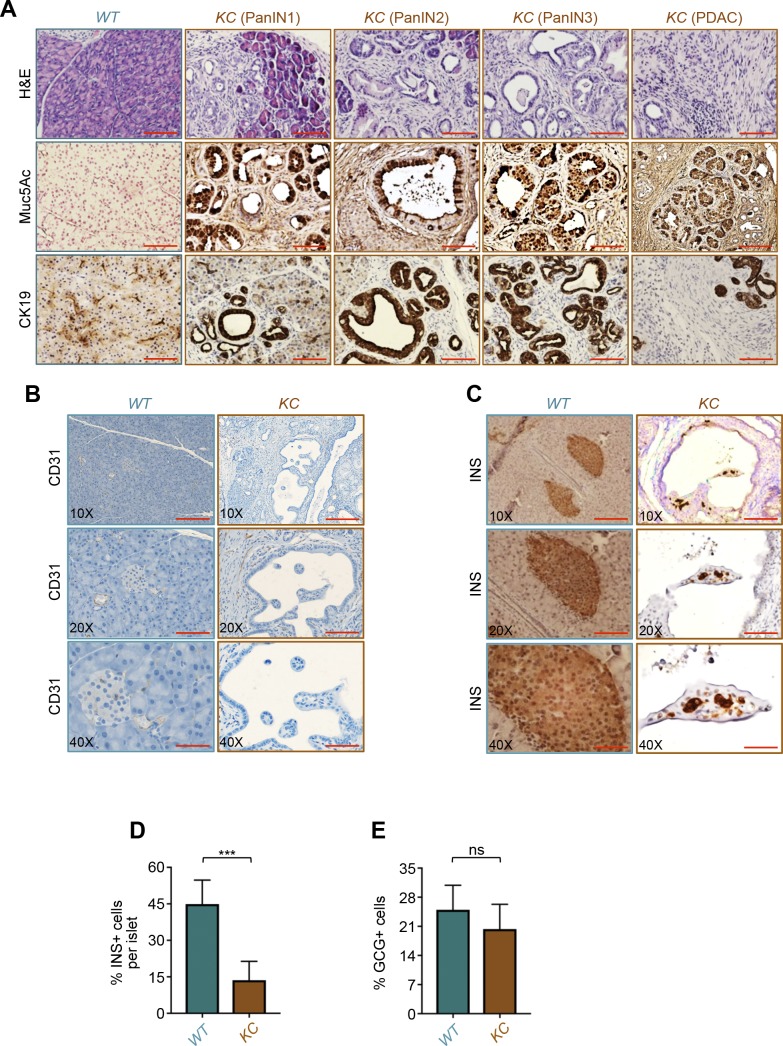
Characterization of PDAC in *KC* mice. **(A)** Formalin-fixed paraffin-embedded (FFPE) sections from *KC* or control (*WT*) mice were stained with H&E or subjected to immunohistochemistry analysis using antibodies to Muc5AC and CK19. Representative pictures of normal pancreas, PanIN1, PanIN2, PanIN3, and full-blown PDAC are shown. Bar, 200 μM. **(B, C)** FFPE sections from *KC* or control mice were subjected to immunohistochemistry analysis using antibodies to CD31 (B) or insulin (C). Representative pictures of normal or cancerous tissues with remnant islets taken at different magnifications are shown. Bar, 400 μM (top), 200 μM (middle), and 100 μM (bottom). **(D, E)** FFPE sections from *KC* or control mice (n = 6) were immunostained with antibodies to insulin or glucagon and revealed by immunofluorescence. Insulin-positive (INS+) or glucagon-positive (GCG+) cells in all islets from six different sections were counted, and results are presented as percentage of INS+ or GCG+ cells relative to the total cell number in islets. Bar, 200 μM. Data are expressed as mean ± SEM. Statistical significance was estimated by unpaired *t* test. ***P < 0.001; ns, nonsignificant.

**Figure 1.**
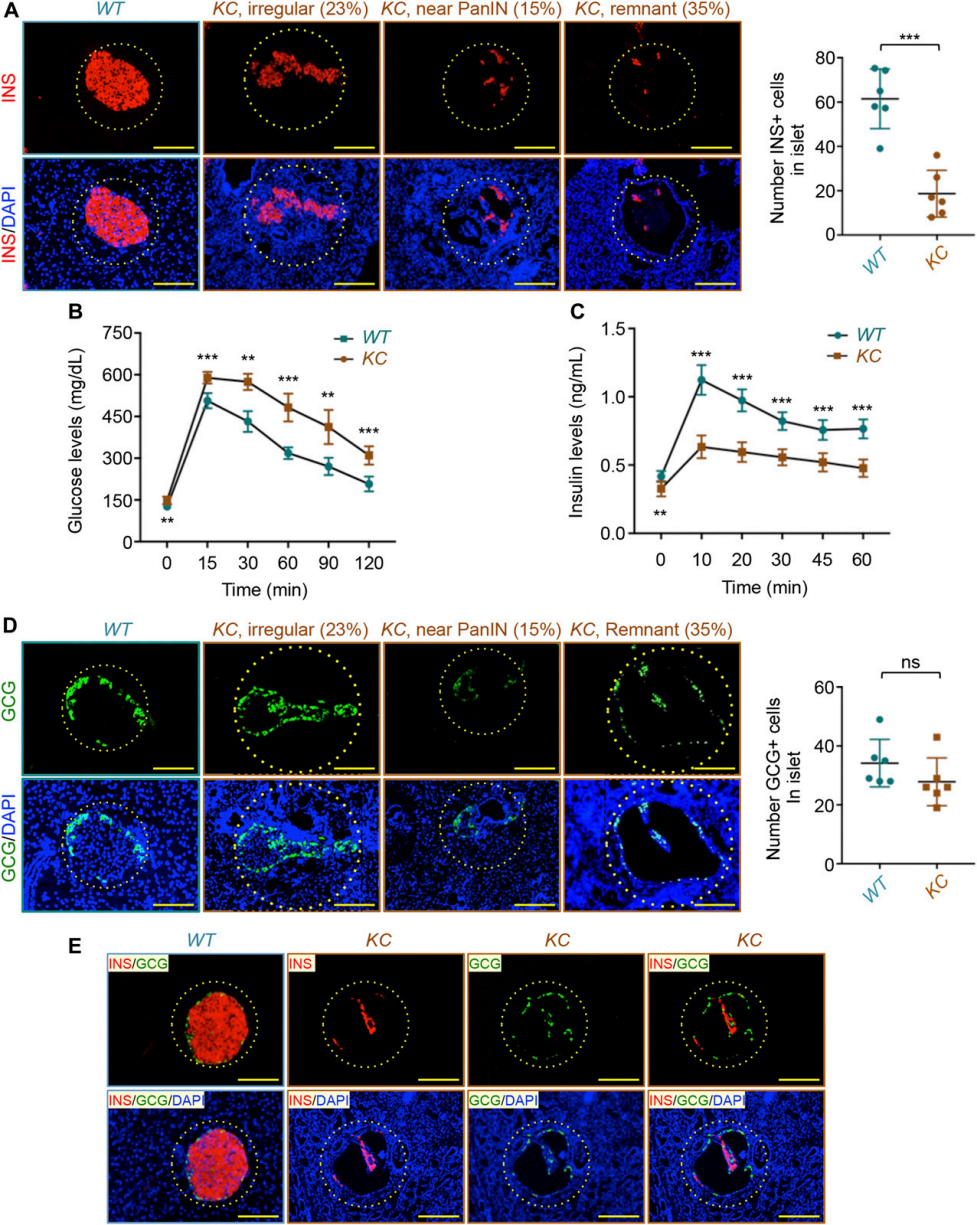
Pancreatic ductal adenocarcinoma induces remnant islet formation. **(A)** Formalin-fixed paraffin-embedded (FFPE) sections from *KC* or control (WT) mice (n = 6) were immunostained with anti-insulin antibody and revealed by immunofluorescence (IF) (red). Nuclei were counterstained with DAPI (blue). Representative pictures of normal, irregular, or remnant islets are shown (left). Percentage of irregular, near-PanIN, or remnant islets is shown. Insulin-positive cells (INS+) in all islets from six different sections were counted, and results are presented as mean of the total number of INS+ cells per islet (right). Bar, 200 μM. **(B)** Glucose tolerance test. Fasted *KC* or control mice were injected with glucose (2 g/kg BW), and blood glucose levels were measured at different intervals during a period of 120 min (n = 6). **(C)** Plasma insulin levels in *KC* and control mice during glucose tolerance test were measured by ELISA (n = 6). **(D)** FFPE sections from *KC* or control mice (n = 6) were immunostained with anti-glucagon antibody and revealed by IF. Representative pictures of normal, irregular, or remnant islets are shown (left). Percentage of irregular, near-PanIN, or remnant islets is shown. Glucagon-positive (GCG+) cells in all islets from six different sections were counted, and results are presented as mean of the total numbers of GCG+ cells per islet (right). Bar, 200 μM. **(E)** FFPE sections from *KC* or control mice were coimmunostained with anti-glucagon and anti-insulin antibodies and revealed by IF. Representative pictures of normal or remnant islets are shown. Bar, 200 μM. For (A, D), data are expressed as dot plot with a line at the median and whiskers showing SD. Statistical significance was estimated by unpaired *t* test. For (B, C), data are expressed as mean ± SEM. Statistical significance was estimated by two-way ANOVA. ***P < 0.001; **P < 0.01; ns, nonsignificant.

To investigate whether PDAC could affect pancreas endocrine functions, we used the *KC* mouse model of PDAC, which faithfully mimics the PanIN to PDAC progression observed in the human disease (Hingorani et al, 2003; Tuveson et al, 2004). This model relies on the *Pdx1-Cre* strain to generate a pancreas-specific expression of a latent endogenous oncogenic *Kras* allele, *LSL-Kras*^*G12D*^. In this model, *Pdx1-Cre* drives expression of Kras^G12D^ in all pancreatic cells, including duct, acinar, and islet cells. In keeping with previous studies (Hingorani et al, 2003; Tuveson et al, 2004), analysis of pancreatic sections from 6- to 12-mo-old *KC* mice stained with hematoxylin and eosin (H&E) or immunostained with antibodies to the ductal marker Cytokeratin 19 (CK19) or Mucin 5Ac (Muc5Ac) showed the presence of various tumor lesions, including PanIN-1, PanIN-2, and PanIN-3 as well as full-blown PDAC (Fig S1A). Perhaps surprisingly, immunofluorescence (IF) staining of pancreatic sections using anti-insulin antibody revealed dramatic alterations in the morphology of the islets, such as the emergence of empty areas within the center of islets which were often situated close but not necessarily adjacent to the tumor areas (Fig 1A). These structures are unlikely to correspond to vascular lumen, as assessed by immunohistochemistry (IHC) using anti-CD31 antibody (Fig S1B). Besides islets with empty areas, we also noticed the presence of irregular islets with distorted shapes, a phenomenon mainly attributed to the compression of the islets by the neighboring tumor lesions (Fig 1A). Similar results were obtained when pancreatic sections were analyzed by IHC using anti-insulin antibody (Fig S1C). To substantiate this finding, we performed glucose tolerance tests using 6-mo-old mice, age at which a significant proportion of *KC* mice develop PanINs and occasionally small full-blown PDAC lesions. As shown in Fig 1B, *KC* mice displayed severe glucose intolerance when compared with control littermates. Consistently, glucose administration was much less efficient at inducing insulin secretion in *KC* mice as compared with control mice (Fig 1C). As such, these findings provide initial hints that PDAC progression might affect the integrity of the islets, which could conceivably lead to impaired glucose tolerance and attendant diabetes.

Because the islets with empty areas in *KC* mice display similar shapes as PanINs, we set out to further explore the exact nature of these new islet-like structures. Taking advantage of abundant literature that α-cells localize mainly to the periphery of the islets, we performed IF experiments using anti-glucagon antibody, with the assumption that the islet-like structures in *KC* mice would also contain α-cells at their periphery if they correspond to islets instead of PanINs. In fact, we consistently detected glucagon-positive cells at the periphery of hollow structures containing very few cells in their central area (Fig 1D). To provide further evidence that these hollow structures correspond to islet-like structures, we observed earlier, we conducted co-IF experiments using antibodies to insulin and glucagon and detected many β-cells at the center of the hollow structures that also contains α-cells at their periphery (Fig 1E). For simplicity, these hollow structures will be referred to hereafter as remnant islets. In efforts to corroborate our finding, we quantified the numbers of insulin- and glucagon-positive cells, and the results revealed a marked decrease in β-cell number in remnant islets in *KC* mice relative to normal islets in control mice (Fig 1A). That decrease was mirrored by a marked reduction in the percentage of β-cells in the total islet cells (Fig S1D). In contrast to β-cells, there was no significant reduction in the number or percentage of α-cells in remnant islets in *KC* mice relative to regular islets in control mice (Figs 1D and S1E). Collectively, these data provide compelling evidence that PDAC drives the formation of remnant islets, perhaps owing to selective depletion of β-cells.

**Figure 2.**
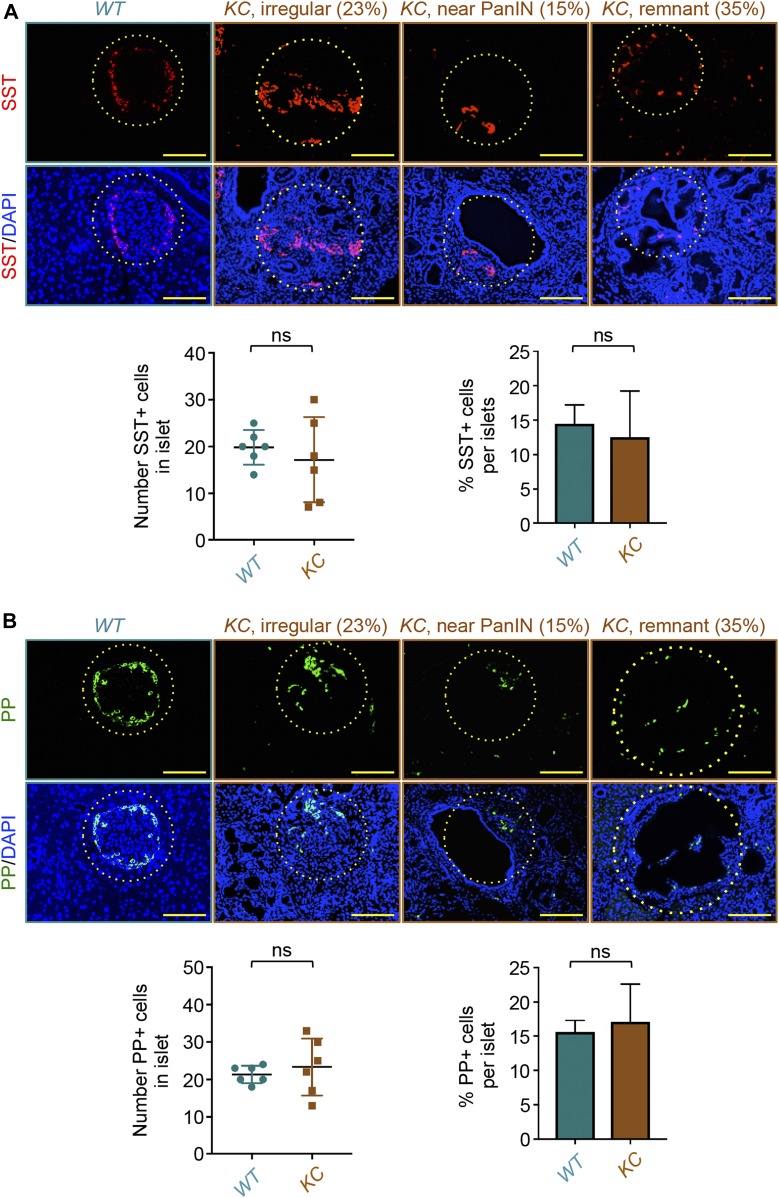
Pancreatic ductal adenocarcinoma triggers selective depletion of β-cells. **(A, B)** Formalin-fixed paraffin-embedded sections from *KC* or control mice (n = 6) were immunostained with anti-somatostatin (SST) or anti-pancreatic polypeptide (PP) antibodies and revealed by immunofluorescence. Representative pictures of normal, irregular, or remnant islets are shown (left). Percentage of irregular, near-PanIN, or remnant islets is shown. Somatostatin-positive (SST+) or pancreatic polypeptide–positive (PP+) cells in all islets from six different sections were counted, and results are presented as mean of the total number of SST+ or PP+ cells per islet (right) or as percentage of SST+ or PP+ cells relative to the total cell number in islets (right). Bar, 200 μM. **(A, B)** For the left graphs in (A, B), data are expressed as dot plot with a line at the median and whiskers showing SD. **(A, B)** For the right graphs in (A, B), data are expressed as mean ± SEM. Statistical significance was estimated by unpaired *t* test; ns, nonsignificant.

Pancreatic endocrine cells other than β-cells, which also localize to the periphery of the islet, play prominent roles in maintaining body glucose homeostasis. For instance, glucagon counteracts the ability of insulin to stimulate gluconeogenesis and glucose release by the liver, whereas somatostatin acts directly on β-cells to suppress insulin secretion (Jain & Lammert, 2009). To corroborate the existence of remnant islets during PDAC progression in *KC* mice, we conducted IF assays using antibodies to somatostatin (SST) and PP to examine whether δ- and PP-cells could also be found at the periphery of the hollow structures. Similar to our earlier observation with α-cells, we detected both δ- and PP-cells at the periphery of hollow structures exclusively in *KC* mice (Fig 2A and B), providing further support to the hypothesis that they might correspond to remnant islets. Quantification of SST- and PP-positive cells failed to show any significant difference in their number or percentage in remnant islets from *KC* mice as compared with normal islets from control mice (Fig 2A and B). Together, these findings illustrate that PanIN and PDAC genesis induces selective erosion of islet β-cell mass and attendant remnant islet formation, thereby likely leading to defective insulin secretion and glucose intolerance. Interestingly, because α and δ cells appeared to be insensitive to PanIN and PDAC, selective depletion of β-cells is expected to create an imbalance in the production of endocrine hormones antagonistic to insulin versus insulin, which could conceivably exacerbate the diabetic complication associated with PDAC.

### Kras^G12D^ drives formation of remnant islets in a cell non-autonomous manner

**Figure 3.**
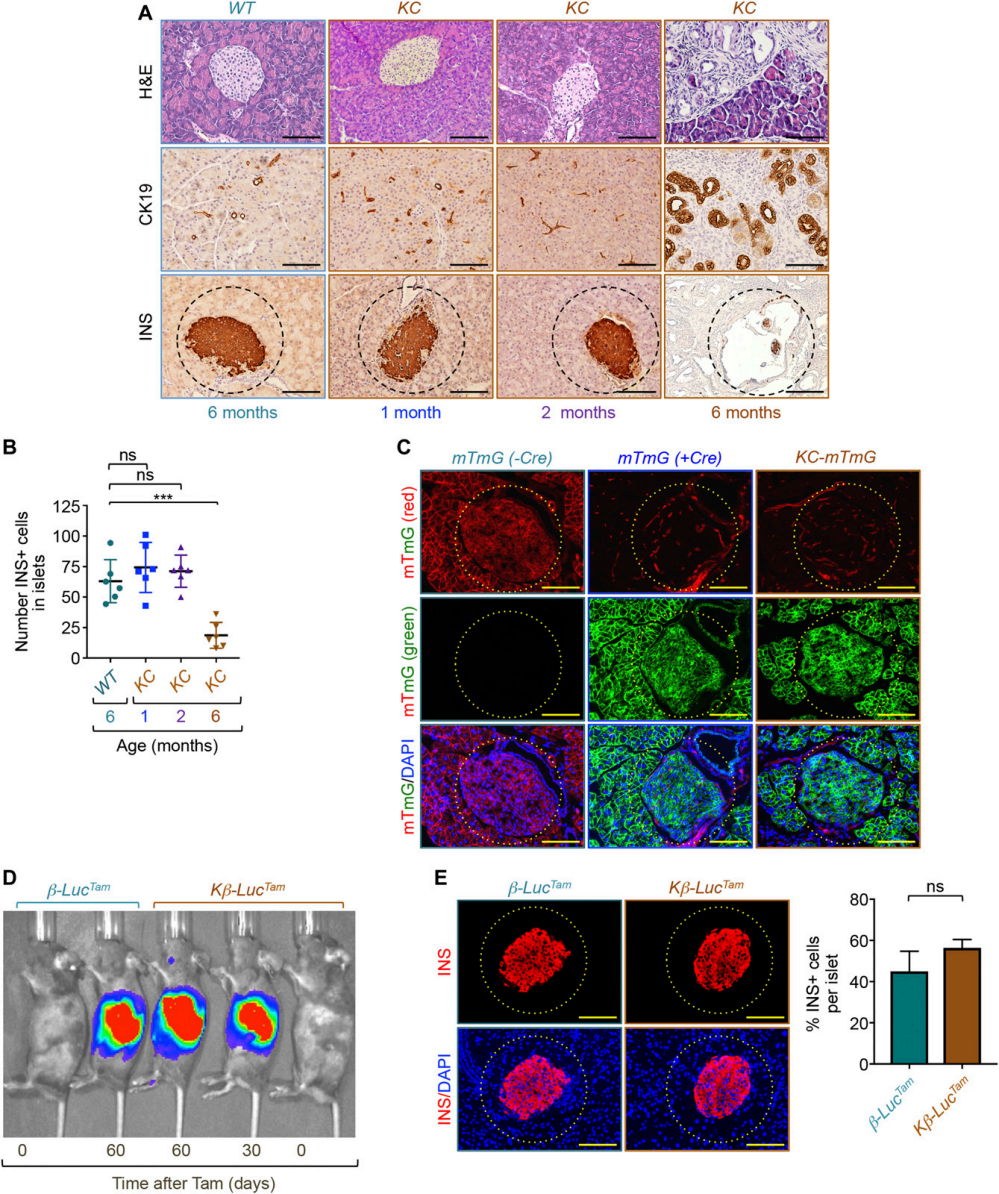
Kras^G12D^ expression in β-cells is dispensable for pancreatic ductal adenocarcinoma–driven remnant islet formation. **(A, B)** Formalin-fixed paraffin-embedded sections from control or *KC* mice (n = 6) at different ages were stained with H&E or immunostained with antibodies to insulin or CK19 and revealed by immunohistochemistry. **(A)** Representative pictures of normal tissues, pancreatic ductal adenocarcinoma lesions, or islets are shown (A). **(B)** INS+ cells in all islets from six different sections were counted, and results are presented as mean of the total number of INS+ cells per islet (B). Bar, 200 μM. **(C)** Frozen pancreatic sections from *KC-mTmG*, *mTmG* (+Cre), or control (−*Cre*) mice (n = 6) were analyzed for GFP (green) or TdTomato (red) fluorescence. Representative pictures of normal islets in *KC-mTmG*, *mTmG*, or control mice are shown. Bar, 100 μM. **(D, E)** 1-mo-old *Kβ-Luc*^*Tam*^ *or* β*-Luc*^*Tam*^ (control) mice (n = 6) were treated with tamoxifen (Tam) and subject to analysis of islet integrity at the age of 6 mo. **(D)** Luciferase activity was analyzed in vivo using the bioluminescence in vivo imaging system (D). **(E)** Formalin-fixed paraffin-embedded sections were immunostained with anti-insulin antibody and INS+ cells in all islets from six different sections were counted, and results are presented as percentage of INS+ cells relative to the total cell number in islets (E). Bar, 200 μM. For (B), data are expressed as dot plot with a line at the median and whiskers showing SD. Statistical significance was estimated by one-way ANOVA. For (E), data are expressed as mean ± SEM. Statistical significance was estimated by unpaired *t* test. ***P < 0.001; ns, nonsignificant.

**Figure S2.**
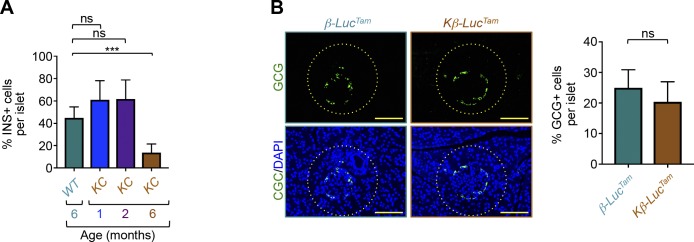
Inability of Kras^G12D^ to drive β-cell depletion. **(A)** Formalin-fixed paraffin-embedded sections from control (6 mo) or *KC* mice (n = 6) at different ages (1–6 mo) were subjected to immunohistochemistry analysis using anti-insulin antibody. INS+ cells in all islets from six different sections were counted and results are presented as percentage of INS+ cells relative to the total cell number in islets. Bar, 200 μM. **(B)** *Kβ-Luc*^*Tam*^ *or* β*-Luc*^*Tam*^ (control) mice were treated with tamoxifen (Tam) and analyzed for islet integrity at the age of 6 mo. Six formalin-fixed paraffin-embedded sections were immunostained with anti-glucagon antibody, quantified for the number of GCG+ cells in all islets, and results are presented as percentage of GCG+ cells relative to the total cell number in islets. Bar, 200 μM. For (A, B), data are expressed as mean ± SEM. **(A, B)** Statistical significance was estimated by one-way ANOVA (A) or unpaired *t* test (B). ***P < 0.001; ns, nonsignificant.

The *Pdx1-Cre* strain used to generate *KC* mice is expressed in pancreatic progenitor cells that give rise to islet, acinar, and ductal compartments (Hingorani et al, 2003; Tuveson et al, 2004). Thus, it is possible that depletion of β-cells in *KC* mice might occur as a consequence of PDAC formation or, alternatively, as a result of Kras^G12D^ expression in β-cells. We undertook several experimental approaches to discriminate between these two possibilities. First, we performed comparative experiments using pancreatic tissues from *KC* mice at different ages (1, 2, and 6 mo) and control mice (6 mo). Consistent with previous studies (Hingorani et al, 2003; Tuveson et al, 2004), we were not able to detect any PanIN or PDAC lesions at the age of 1 or 2 mo, as gauged by both H&E staining and IHC using anti-CK19 antibody (Fig 3A). At this age, *KC* mice did not exhibit any irregular or remnant islets (Fig 3A), suggesting that Kras^G12D^ expression in islets might not be responsible for the depletion of β-cells in *KC* mice. Of note, we detected a significant number of remnant islets in 6-mo-old *KC* mice, age at which those mice began to develop PanIN and occasionally PDAC lesions (Fig 3A). We confirmed these results by determining the number and percentage of insulin-positive cells within the islets (Figs 3B and S2A). Second, to provide further evidence that Kras^G12D^ expression in β-cells is not sufficient to drive remnant islet formation, we sought to conduct a lineage tracing strategy using a double-fluorescent system that allows for the evaluation of both recombined and non-recombined cells within the same tissue (Muzumdar et al, 2007). For this, we crossed our *KC* mice with *ROSA*^*mT-mG*^ mice, which express membrane-localized TdTomato in a widespread fashion before Cre recombinase exposure, and membrane-localized GFP after recombination (Muzumdar et al, 2007). Although most of the islets were positive for GFP and negative for TdTomato in 2-mo-old *KC* mice, the vast majority of them were completely intact (Fig 3C), confirming the inability of Kras^G12D^ to induce β-cell depletion when expressed in islet cells. Third, to demonstrate directly that expression of Kras^G12D^ per se is not able to drive β-cell depletion in a cell autonomous manner, we crossed *LSL-Kras*^*G12D*^ mice with a tamoxifen-inducible Cre recombinase (*Ins2-Cre*^*ERT2*^) to drive expression of Kras^G12D^ specifically in β-cells (Wang et al, 2012). To monitor the recombination in these mice in vivo, we also included a Cre-activable allele of luciferase (*LSL-Luc*) knocked into the Rosa26 locus (Cheung et al, 2008). We choose to activate Cre recombinase in 1-mo-old mice and analyze islets integrity at 6 mo, age at which a significant number of *KC* mice develop PanIN/PDAC lesions and associated remnant islets. Bioluminescence imaging in vivo showed robust luciferase activity after tamoxifen injection (Fig 3D), indicative of efficient recombination. Remarkably, IF using anti-insulin or anti-glucagon antibodies failed to reveal any alteration in islet β-cells (Figs 3E and S2B), arguing against the possibility that Kras^G12D^ expression directly affects β-cell mass. Collectively, these findings strongly suggest that PanIN and/or PDAC formation drives β-cell depletion and remnant islet formation in a cell non-autonomous manner.

### Specificity of PDAC-driven remnant islet formation

**Figure S3.**
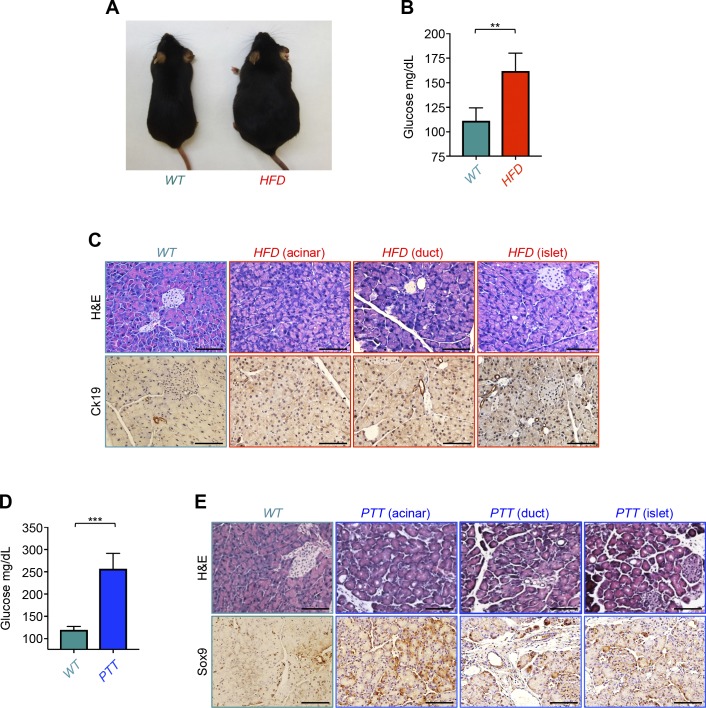
Characterization of pancreatic tissues from mice receiving high-fat diet (HFD) or caerulein. **(A, B, C)** Control mice (n = 6) were fed HFD and subjected to obesity and diabetes analyses after 3 mo. **(A)** Representative of normal or obese mice are shown (A). **(B)** Mice were fasted for 6 h and analyzed for blood glucose (B). Formalin-fixed paraffin-embedded sections were stained with H&E or subjected to immunohistochemistry analysis using anti-CK19 antibody. **(C)** Representative pictures of acinar, ductal, and islet compartments are shown (C). **(D, E)** Control mice (n = 6) were injected with caerulein and subjected to diabetes analyses after 2 d of treatment. **(D)** Mice were fasted for 6 h and analyzed for blood glucose (D). Formalin-fixed paraffin-embedded sections were stained with H&E or subjected to immunohistochemistry analysis using anti-Sox9 antibody. **(E)** Representative pictures of acinar, ductal, and islet compartments are shown (E). Bar, 200 μM. For (B, D), data are expressed as mean ± SEM. Statistical significance was estimated by unpaired *t* test. **P < 0.01; ***P < 0.001.

**Figure S4.**
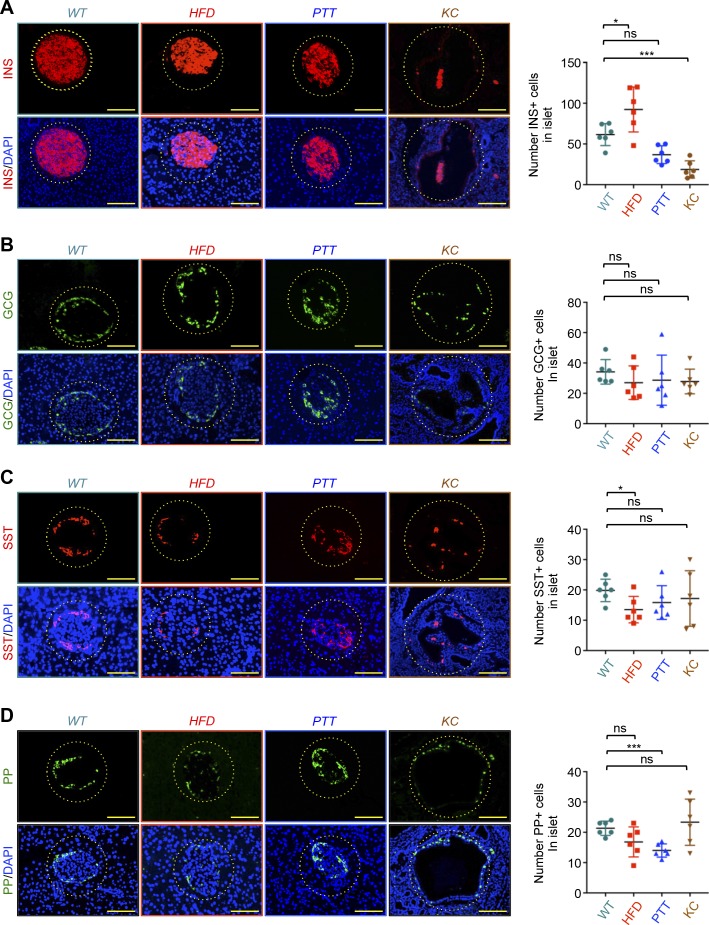
Specificity of pancreatic ductal adenocarcinoma–driven β-cell depletion. **(A, B, C, D)** Formalin-fixed paraffin-embedded sections from control, pancreatitis (PPT), HFD-fed (HFD), or *KC* mice (n = 6) were immunostained with antibodies to insulin, glucagon, somatostatin, or PP and revealed by immunofluorescence. Representative pictures of normal or remnant islets are shown (left). INS+, GCG+, SST+, or PP+ cells in all islets from six different sections were counted, and results are presented as mean of the total number of hormone-positive cells per islet (right). Bar, 200 μM. Data are expressed as dot plot with a line at the median and whiskers showing SD. Statistical significance was estimated by two-way ANOVA. *P < 0.05; ***P < 0.001; ns, nonsignificant.

Obesity-associated diabetes has been shown to occur either as a result of insulin resistance in peripheral tissues or β-cell failure due to inflammation (Donath et al, 2013). In addition, obesity represents a major risk factor for the development of PDAC (Malhi & Camilleri, 2017). To understand further the mechanisms leading to diabetes in PDAC, we conducted comparative experiments using *KC* mice and age-matched wild-type mice rendered obese and diabetic (hyperglycemia) by HFD feeding for 24 wk (Fig S3A and B). In contrast to *KC* mice, these obese mice maintained normal islet morphology and β-cell mass and did not develop any remnant islets or any other alterations, as evidenced by both H&E staining and insulin or CK19 immunostaining (Figs S3C and 4A). We also observed normal distribution and localization of other pancreatic islet cells, including α, δ, and PP cells (Fig S4B–D). Quantification of islet cells showed a significant increase in the number of insulin-positive cells in HFD mice compared with control mice (Fig S4A), consistent with previous studies (Morioka et al, 2007; Fu et al, 2009). In contrast, there were minor or no significant differences in the numbers of α, δ, or PP cells (Fig S4B–D). Based on the literature and our findings (Donath et al, 2013; Malhi & Camilleri, 2017), it seems unlikely that obesity facilitates PDAC pathogenesis by causing selective depletion of islet β-cells.

**Figure 4.**
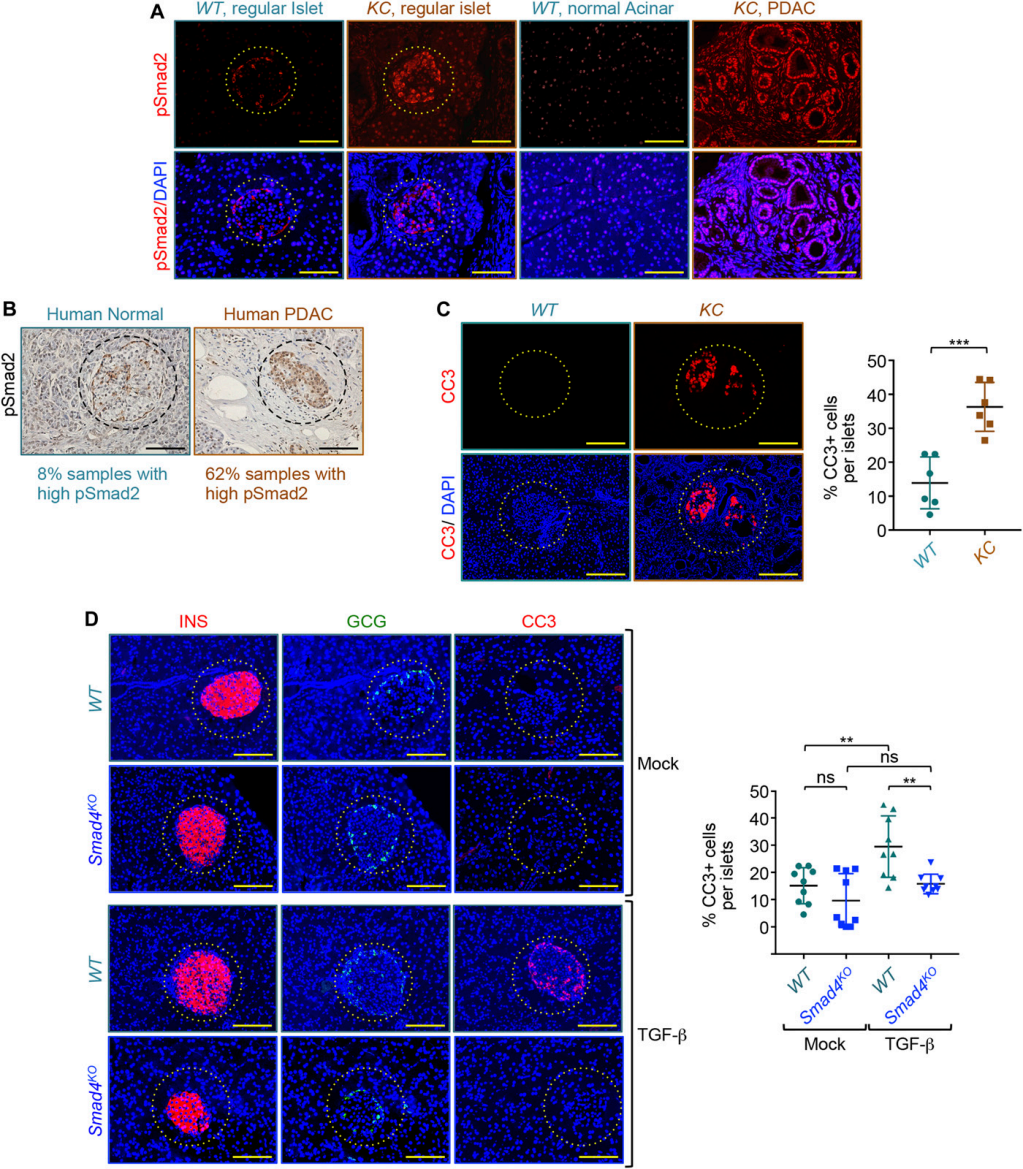
Activation of TGF-β signaling induces β-cell death. **(A)** Formalin-fixed paraffin-embedded sections from *KC* or control mice (n = 6) were immunostained with anti–phopspho-Smad2 antibody (pSmad2) and revealed by immunofluorescence (IF). Representative pictures of islets and pancreatic ductal adenocarcinoma (PDAC) areas are shown. Bar, 200 μM. **(B)** Human tissue microarrays containing normal or PDAC tissues (n = 48) were immunostained with anti-pSmad2 antibody and revealed by immunohistochemistry. Representative pictures of islets in normal and PDAC tissues are shown. Percentage of normal and PDAC samples with high pSmad2 is shown. Bar, 200 μM. **(C)** Formalin-fixed paraffin-embedded sections from *KC* or control mice (n = 6) were immunostained with anti–cleaved caspase 3 antibody (CC3) and revealed by IF. Representative pictures of islets and PDAC areas are shown (left). CC3+ cells in all islets from six different sections were counted, and results are shown as percentage of CC3+ cells relative to the total cell number in islets (right). Bar, 200 μM. **(D)** *Smad4*^*KO*^ or control mice (n = 3) were treated with TGF-β1 for 3 d and expression of insulin (red), glucagon (green), or CC3 (red) was analyzed by IF (left). CC3+ cells in all islets from six different sections were counted, and results are presented as percentage of CC3+ cells relative to the total cell number in islets (right). Bar, 200 μM. For (C, D), data are expressed as dot plot with a line at the median and whiskers showing SD. **(C, D)** Statistical significance was estimated by unpaired *t* test (C) or two-way ANOVA (D). ***P < 0.001; **P < 0.01; ns, nonsignificant.

Pancreatitis is caused by pancreatic inflammation that results in progressive nutrient maldigestion, which leads to severe metabolic imbalance (Ewald & Hardt, 2013). Commonly experienced alongside pancreatitis is diabetes, which occurs because of the obliteration of islet cells caused by intrapancreatic inflammation (Ewald & Hardt, 2013; Kleeff et al, 2017). Moreover, there is abundant evidence that inflammation associated with pancreatitis promotes PDAC development in both human and murine models, which promoted us to explore whether pancreatitis could trigger the formation of remnant islets (Su et al, 2006; Guerra et al, 2011). The most widely used model of pancreatitis involves treatment with the cholecystokinin agonist caerulein, which induces local oxidative stress, inflammation, edema, and loss of the acinar parenchyma that is transiently replaced by a duct-like epithelium, features reminiscent of human pancreatitis (Su et al, 2006; Guerra et al, 2011). As anticipated, fasting blood glucose was higher in mice injected with caerulein than those injected with vehicle (Fig S3D). Interestingly, H&E staining and Sox9 (a prominent marker of pancreatitis) immunostaining of pancreatic tissues indicated that pancreatitis affected the acinar compartment, but did not cause formation of remnant islets (Figs S3E and S4A–D). We independently confirmed this observation in IF experiments using antibodies to insulin, glucagon, somatostatin, and PP (Fig S4A–D). As anticipated, pancreatic tissues from age-matched *KC* mice harbor many remnant islets (Fig S4A–D). Collectively, these findings indicate that pancreatitis does not lead to significant disorganization of islets or cause depletion of β-cells, as does PDAC.

### Activation of TGF-β signaling in islets during PDAC progression

**Figure S5.**
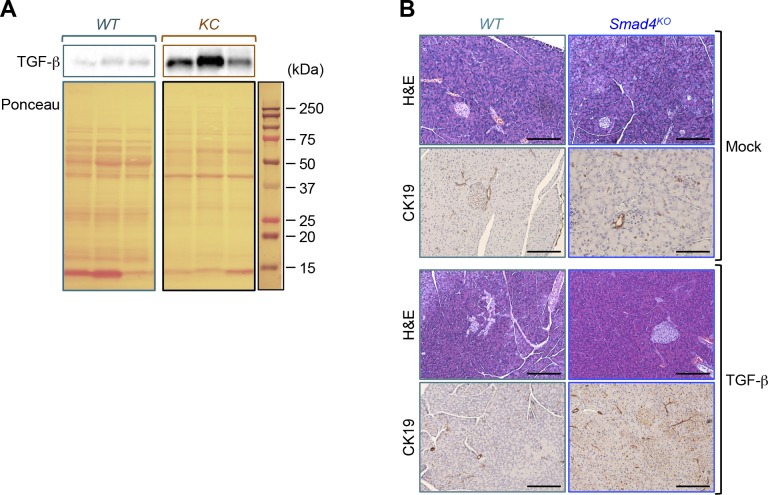
Activation of TGF-β signaling in pancreatic ductal adenocarcinoma. **(A)** Equal amounts of pancreatic protein extracts from *KC* or control mice were subjected to immunoblotting analyses using antibodies to TGF-β1. Immunoblotting pictures for WT and KC mice were obtained from the same membrane. Because of variations in the expression of housekeeping genes (α-actin and GAPDH) between individual mice, we used red ponceau staining to verify the loading. **(B)** Formalin-fixed paraffin-embedded sections from control or *Smad4*^*KO*^ mice (n = 3) were stained with H&E or subjected to immunohistochemistry analysis using anti-CK19 antibody. Representative pictures of pancreatic tissues are shown. Bar, 200 μM.

The fact that PanIN and PDAC formation within the exocrine pancreatic epithelium can trigger β-cell depletion within the islets suggests the existence of a paracrine factor produced by cancer cells or their supporting stroma that acts on islets to trigger depletion of β-cells. Among the prominent growth factors that are highly produced during PDAC progression is TGF-β, which is known to be associated with poor outcome in PDAC patients (Friess et al, 1993; Bardeesy et al, 2006). We confirmed the increase in TGF-β production in pancreatic tissues from *KC* mice by immunoblotting (Fig S5A). Of particular importance, previous studies have shown that inhibition of TGF-β facilitates human islet transplantation (Xiao et al, 2016). In addition, activation of Smad signaling has been shown to induce apoptosis or inhibit replication of β-cell in vitro (Zhao et al, 2012; Dhawan et al, 2016), further emphasizing TGF-β as an attractive candidate that might mediate β-cell depletion during PDAC progression. To explore this possibility, we first analyzed the phosphorylation of Smad2 on S465/S467 (pSmad2), which is directly catalyzed by the activated TGF-β receptor complex (Massague et al, 2005). IF experiments showed a marked increase in pSmad2 in both PanIN and PDAC lesions (Fig 4A), which is in agreement with previous studies (Friess et al, 1993; Bardeesy et al, 2006). Besides these cancerous areas, we also detected strong phosphorylation of Smad2 in normal adjacent tissues as well as in islets before β-cell depletion (Fig 4A), indicative of general activation of TGF-β signaling in the pancreatic epithelium harboring PanIN and PDAC lesions. A similar increase in Smad2 phosphorylation was also observed in islets within human PDAC tissues when compared with islets within normal human pancreatic tissues (Fig 4B).

Next, to investigate whether hyperactivation of TGF-β/Smad signaling could drive β-cells apoptosis in PDAC tissues, we performed IF experiments to analyze the expression of cleaved caspase 3 (CC3), a surrogate readout of apoptosis (Kurokawa & Kornbluth, 2009). The experiment depicted in Fig 4C shows a marked increase in CC3 positive (CC+) cells in islets from *KC* mice as compared with islets from control mice. These CC3+ cells are likely to correspond to β-cells as *KC* mice displayed a decrease in β-cell numbers, but not in α, δ, or PP or cell numbers (Figs 1A–E and 2A and B). To determine whether this apoptotic phenotype could be initiated by TGF-β signaling, we injected wild-type mice with TGF-β1 for three consecutive days and subsequently analyzed CC3 accumulation within the islets. Treatment with TGF-β1 did not affect pancreas histology, as assessed by both H&E staining and CK19 immunostaining (Fig S5B). However, we observed a strong accumulation of CC in islets after injection of mice with TGF-β1 (Fig 4D), indicating that increased systemic TGF-β levels is sufficient to drive apoptotic β-cell death. To determine whether this response could be mediated via Smad signaling, we performed similar experiments using mice with pancreas-specific deletion of *Smad4* (*Smad4*^*KO*^), an essential component of TGF-β signaling (Massague et al, 2005). We found that ablation of *Smad4* completely abolished TGF-β–mediated CC3 accumulation within the islets (Fig 4D). Together, these in vivo data revealed an ability of TGF-β signaling to drive apoptotic β-cell death.

### Inactivation of TGF-β signaling suppresses PDAC-driven β-cell depletion

**Figure S6.**
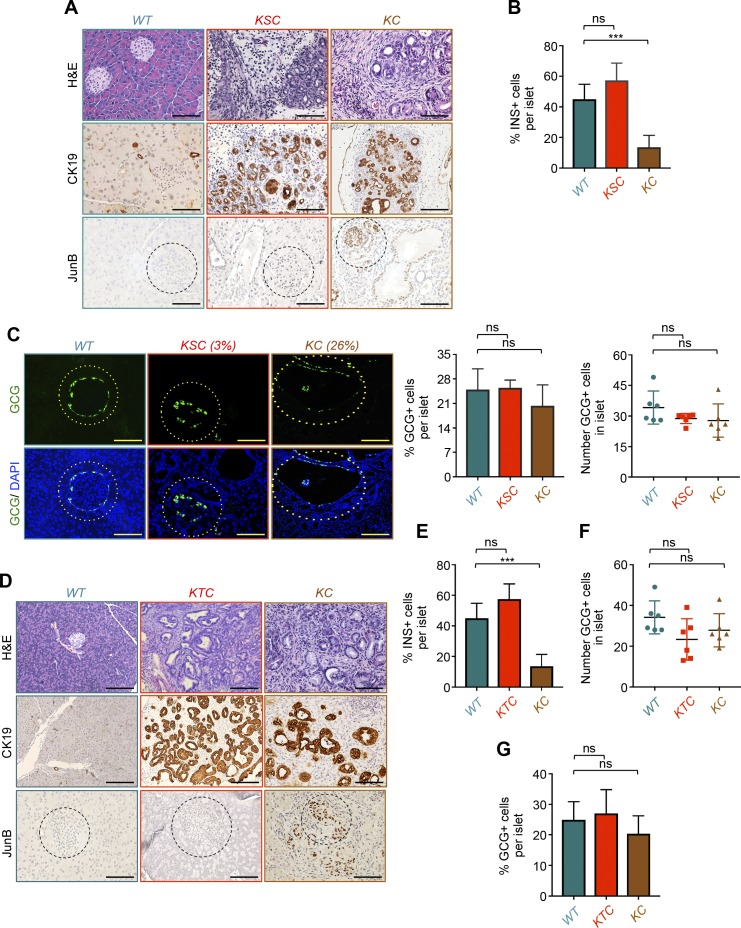
Inactivation of TGF-β signaling suppresses pancreatic ductal adenocarcinoma (PDAC)–driven β-cell depletion. **(A)** Formalin-fixed paraffin-embedded (FFPE) sections from control, *KC*, or *KSC* mice were stained with H&E or subjected to immunohistochemistry analysis using antibodies to CK19 or JunB. Representative pictures of normal or PDAC tissues are shown. Bar, 200 μM. **(B)** FFPE sections from control, *KC*, or *KSC* mice (n = 6) were immunostained with anti-insulin antibody and revealed by immunofluorescence (IF). Then, INS+ cells in all islets from six different sections were counted, and results are presented as percentage of INS+ cells relative to the total cell number in islets. Bar, 200 μM. **(C)** FFPE sections from control, *KC*, or *KSC* mice (n = 6) were immunostained with anti-glucagon antibody and revealed by IF. Representative pictures of normal or remnant islets are shown (left). Percentage of remnant islets is shown. GCG+ cells in all islets from six different sections were counted, and results are presented as percentage of GCG+ relative to the total cell number in islets (middle) or as mean of the total number of GCG+ cells per islet cells (right). Bar, 200 μM. **(D)** FFPE sections from control, *KC*, or *KTC* mice were stained with H&E or immunostained with antibodies to CK19 or JunB and revealed by immunohistochemistry. Representative pictures of normal or PDAC tissues are shown. **(E, F, G)** FFPE sections from control, *KC*, or *KTC* mice (n = 6) were immunostained with antibodies to insulin or glucagon and revealed by IF. **(E, F, G)** Then, INS+ or GCG+ cells in all islets from six different sections were counted, and results are presented as percentage of INS+ or GCG+ cells relative to the total cell numbers in islets (E, F) or as mean of the total number of GCG+ cells per islet cells (G). For (B, C [middle], E, G), data are expressed as mean ± SEM. For (C [right], F), data are expressed as dot plot with a line at the median and whiskers showing SD. For all graphs, statistical significance was estimated by one-way ANOVA. ***P < 0.001; ns, nonsignificant.

**Figure 5.**
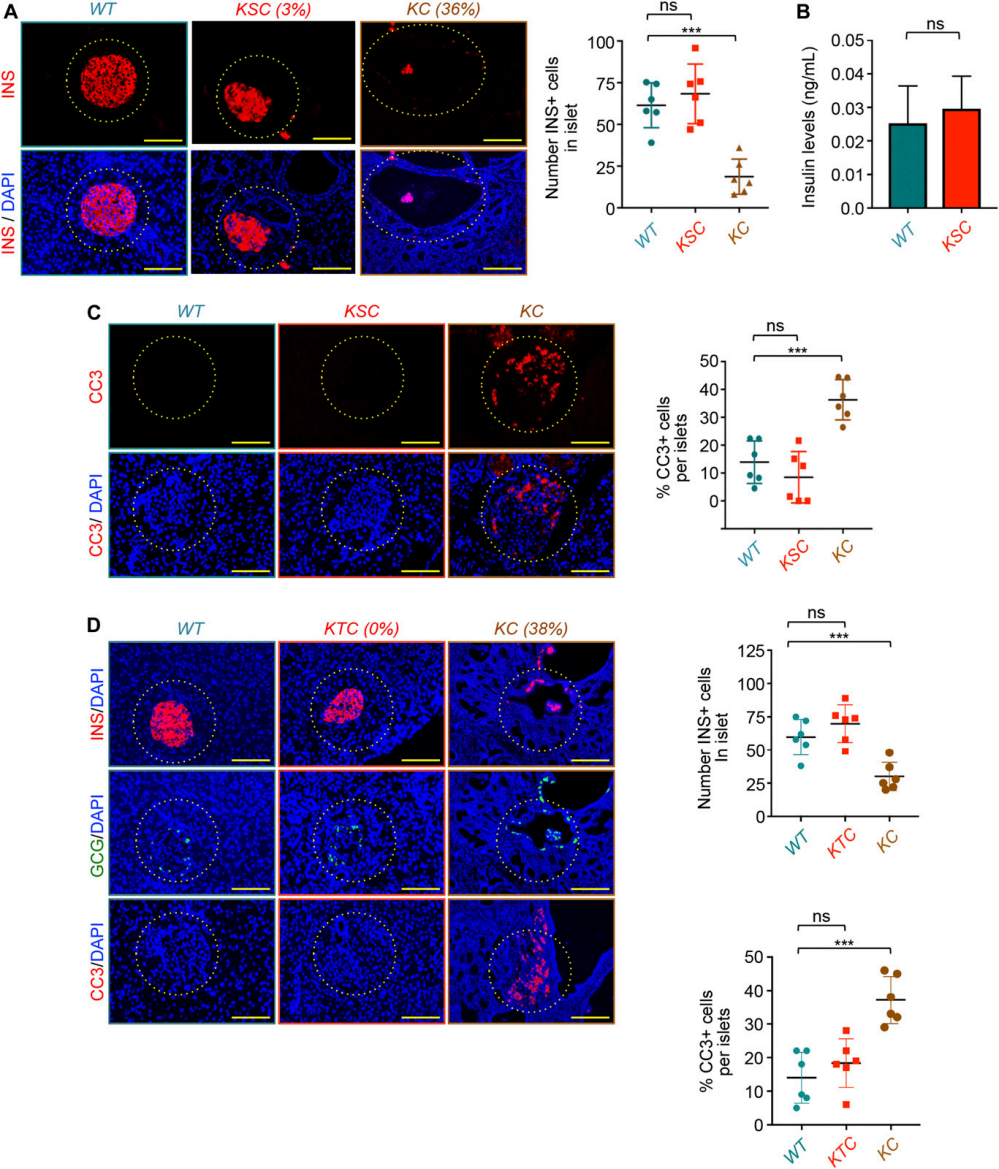
Inactivation of TGF-β signaling suppresses pancreatic ductal adenocarcinoma–induced β-cell depletion. **(A)** Formalin-fixed paraffin-embedded (FFPE) sections from control, *KC*, or *KSC* mice (n = 6) were immunostained with anti-insulin antibody and revealed by immunofluorescence (IF). Representative pictures of normal or remnant islets are shown (left). Percentage of remnant islets is shown. INS+ cells in all islets from six different sections were counted, and results are presented as means of the total number of INS+ cells in islets (right). Bar, 200 μM. **(B)** Plasma insulin levels in control or *KSC* mice (n = 6). **(C)** FFPE sections from control, *KC*, or *KSC* mice (n = 6) were immunostained with anti-CC3 and revealed by IF. Representative pictures of islets are shown (left). CC3+ cells in all islets from six different sections were counted, and results are presented as percentage of CC3+ cells relative to the total cell number in islets (right). Bar, 200 μM. **(D)** FFPE sections from control, *KC*, or *KTC* mice (n = 6) were immunostained with antibodies to insulin, glucagon, or CC3 and revealed by IF. Representative pictures of normal or remnant islets are shown (left). Percentage of remnant islets is shown. INS+ or CC3+ cells in all islets from six different sections were counted, and results are presented as percentage of CC3+ cells relative to the total cell number in islets (right). Bar, 200 μM. For (A [center], C, D), data are expressed as dot plot with a line at the median and whiskers showing SD. Statistical significance was estimated by one-way ANOVA. For (B), data are expressed as mean ± SEM. Statistical significance was estimated by unpaired *t* test. ***P < 0.001; ns, nonsignificant.

To provide further evidence that PDAC induces depletion of β-cells through activation of TGF-β signaling, we generated mice deleted of *Smad4* in a *Kras*^*G12D*^ background (referred to hereafter as *KSC*). Both H&E staining and CK19 immunostaining showed abundant cancerous lesions within the pancreatic tissues of *KSC* mice (Fig S6A). Furthermore, we found that deleting *Smad4* blunted the accumulation of the TGF-β target gene *JunB* (Fig S6A), attesting to the inactivation of TGF-β signaling in these mice. Most importantly, immunostaining with insulin and glucagon antibodies showed almost complete absence of remnant islets in *KSC* mice as compared with *KC* mice (Figs 5A and S6B and C). Quantification of insulin-positive cells showed that *Smad4* ablation in *KSC* mice was able to restore the number and percentage of β-cells to levels approaching those of the wild-type littermates (Figs 5A and S6B). We independently confirmed this observation by measuring blood insulin levels in these mice (Fig 5B). We also extended our experiments to examine the effects of inactivating TGF-β signaling on PDAC-induced β-cell death and found that *Smad4* deletion in *KC* mice was effective at reversing the apoptotic phenotype as well (Fig 5C). Noteworthy, we were not able to see any significant difference in the number or percentage of glucagon-positive cells irrespective of the genetic background, for example, *KC*, *KSC*, and wild-type (Fig S6C).

To further investigate the role of TGF-β signaling in PDAC-associated β-cell depletion, we used mice with the combined expression of *Kras*^*G12D*^ and homozygous deletion of the TGF-β type II receptor (*TβRII, mice* referred to hereafter as *KTC* mice). As for Smad4, deleting *TβRII* resulted in almost complete blockade of JunB accumulation (Fig S6D), indicative of efficient inactivation of TGF-β signaling. Interestingly, despite the presence of abundant PanIN and PDAC lesions within the pancreas of *KTC* mice, there were no apparent alterations in islets, as evidenced by immunostaining with anti-insulin and anti-glucagon antibodies (Figs 5D and S6D–G). Similar results were obtained when islets from *KC* and *KTC* mice were analyzed for apoptotic cell death by CC3 immunostaining (Fig 5D). Quantification of the insulin- and CC3-positive cells confirmed that *TβRII* deletion was able to suppress β-cell death and remnant islet formation (Fig 5D). Based on these findings, we conclude that inactivation of TGF-β signaling is sufficient to prevent depletion of β-cells driven by PanIN and PDAC lesions.

### Translational relevance of targeting TGF-β signaling in PDAC

**Figure S7.**
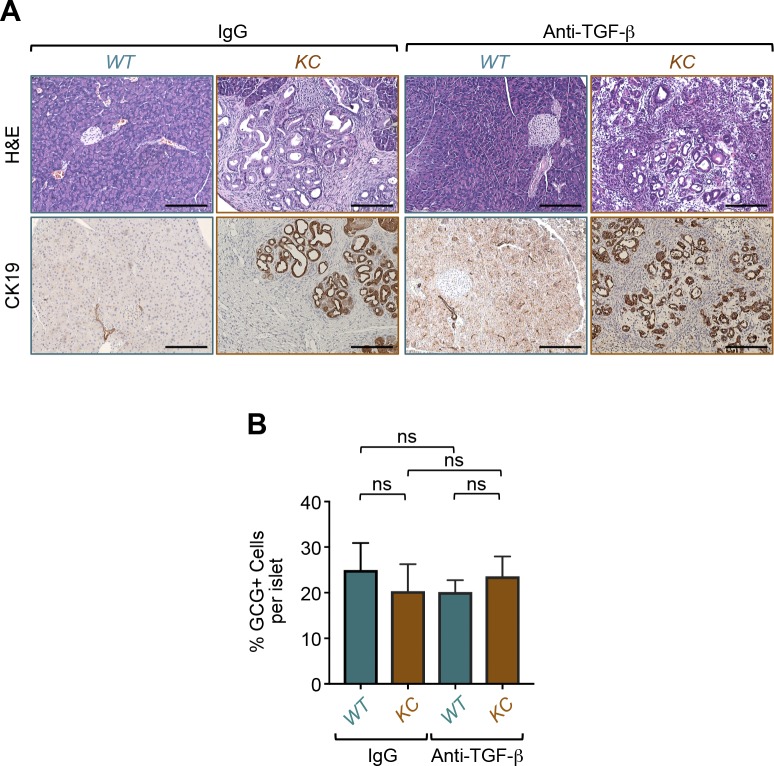
Suppression of TGF-β signaling impedes pancreatic ductal adenocarcinoma–driven β-cell depletion. **(A)** *KC* or control mice (n = 6) were treated with an antibody-targeting TGF-β1-3 or isotype-matched antibody for 4 mo. **(A)** Then, formalin-fixed paraffin-embedded sections were stained with H&E (A) or immunostained with antibodies to CK19 (A) and revealed by immunohistochemistry. Representative pictures of normal or pancreatic ductal adenocarcinoma tissues are shown. **(B)** *KC* or control mice (n = 6) were treated with an antibody-targeting TGF-β1-3 or isotype-matched antibody for 4 mo. Then, formalin-fixed paraffin-embedded sections were immunostained with anti-glucagon antibody and revealed by immunofluorescence. GCG+ positive cells in all islets from six different sections were counted, and results are presented as percentage of GCG+ cells relative to the total cell number in islets. Bar, 200 μM. Data are expressed as mean ± SEM. Statistical significance was estimated by two-way ANOVA; ns, nonsignificant.

**Figure 6.**
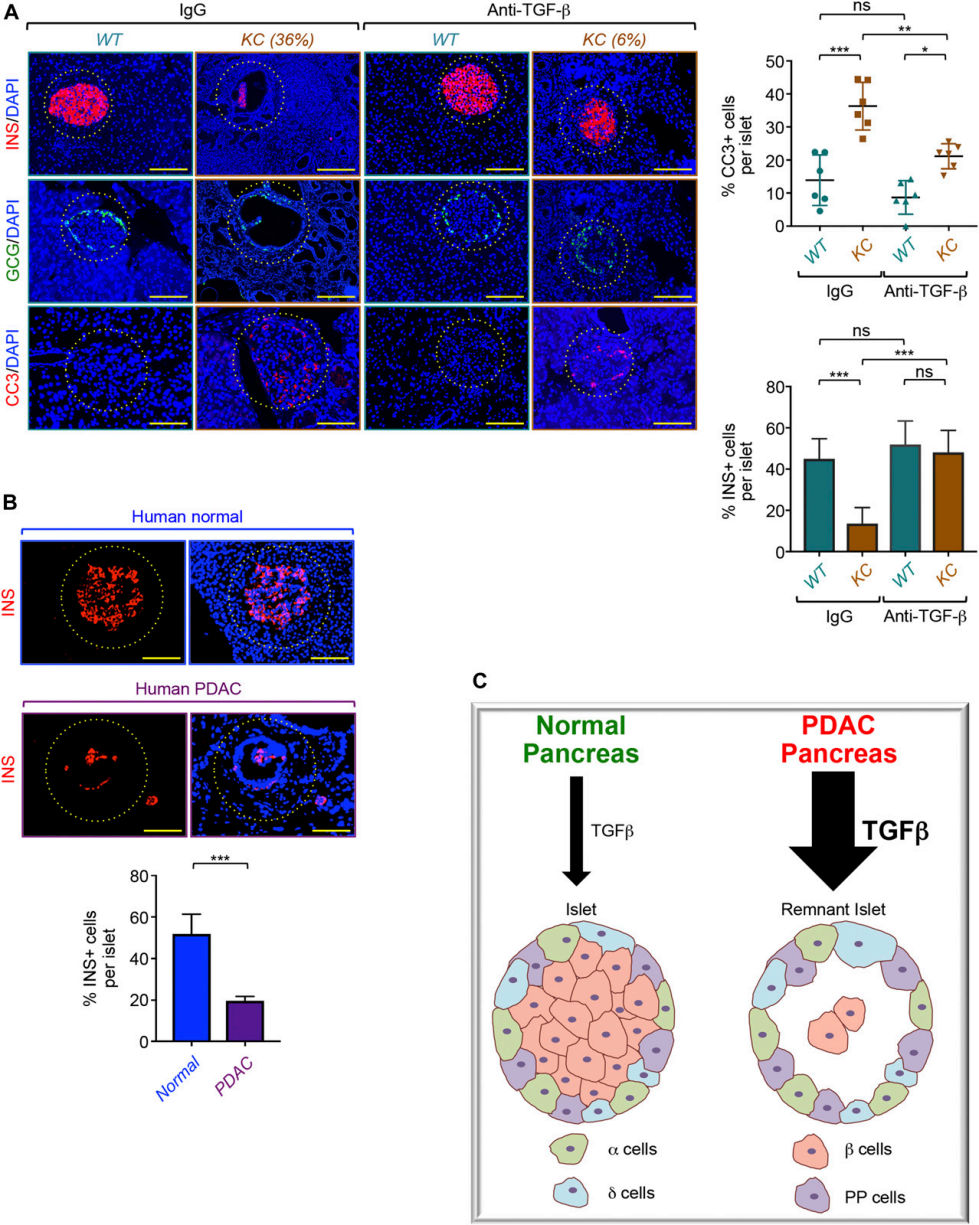
Translational relevance of TGF-β signaling to pancreatic ductal adenocarcinoma (PDAC)–driven remnant islet formation. **(A)** *KC* or control mice (n = 6) were treated with an antibody-targeting TGF-β1-3 or isotype-matched antibody for 4 mo, and the expression of insulin (red), glucagon (green), or CC3 (red) was analyzed by immunofluorescence (left). Representative pictures of normal or remnant islets are shown. Percentage of remnant islets is shown. INS+ or CC3+ cells in all islets from six different sections were counted, and results are presented as percentage of CC3+ or INS+ cells relative to the total cell number in islets (right). Bar, 200 μM. **(B)** Human tissue microarrays containing normal of PDAC tissues (n = 48) were immunostained with antibodies to insulin and revealed by immunofluorescence. Representative pictures of normal islets or remnant islets are shown (top). INS+ cells in all islets found in the tissue microarray (TMA) were counted, and results are presented as percentages of INS+ cells relative to the total cell number in islets (bottom). Bar, 200 μM. **(C)** A model for PDAC-driven remnant islet formation. For (A) (top, right), data are expressed as dot plot with a line at the median and whiskers showing SD. Statistical significance was estimated by two-way ANOVA. For (A [bottom, right], B [bottom]), data are expressed as mean ± SEM. **(A, B)** Statistical significance was estimated by two-way ANOVA (A) or unpaired *t* test (B). *P < 0.05; **P < 0.01; ***P < 0.001; ns, nonsignificant.

To ascertain the translational potential of our findings, we wondered whether suppressing TGF-β signaling in vivo by a pharmacological strategy could preserve β-cell mass under PDAC conditions. Accordingly, we took advantage of the availability of a commercial antibody that neutralizes the most abundant forms of TGF-β: TGF-β1, TGF-β2, and TGF-β3. We designed four groups of control and *KC* mice that we treated with control antibody (IgG) or anti–TGF-β antibody for 4 mo. We began the treatments at the age of 2 mo, time at which most *KC* mice do not display any apparent PanIN lesions (Fig 3A). At necropsy, both H&E staining and CK19 immunostaining showed normal pancreatic tissues in control mice irrespective of whether they were receiving control IgG or anti–TGF-β antibody (Fig S7A). Moreover, we found that *KC* mice treated with anti–TGF-β did not display any significant increase in size or number of PanIN and PDAC lesions (Fig S7A). In marked contrast, anti–TGF-β treatment almost completely blocked the formation of remnant islets, as assessed by immunostaining with insulin and glucagon antibodies (Figs 6A and S7B). A similar conclusion could be drawn when apoptotic β-cell death was examined by CC3 immunostaining (Fig 6A). Thus, immunological suppression of TGF-β signaling can afford substantial protection against PDAC-driven β-cell mass erosion.

To establish further the translation relevance of our findings, we made use of a human tissue microarray containing human PDAC and normal pancreatic tissues. As discussed above, we detected a strong immunoreactivity of pSmad2 within the islets (Fig 4B), consistent with the activation of TGF-β signaling. More importantly, we consistently detected hollow structures that contain a few insulin-positive cells at the center (Fig 6B), reminiscent of remnant islet formation. Quantification of these results showed a marked decrease in the percentage of β-cells per islet (Fig 6B), providing further evidence to the existence of remnant islets within human PDAC tissues. These findings indicate that human PDAC formation can trigger selective depletion of islet β-cells, thus shedding new light into the mechanisms by which this malignancy could drive diabetes.

## Discussion

PDAC is one of the most lethal human malignancies, with a 5-yr survival rate of less than 5% (Hidalgo, 2010). After decades of intense investigations, there are still no effective diagnostic methods available for PDAC patients due in part to the absence of early symptoms, which prevents detection before late stages when the malignancy becomes invasive and intractable (Hidalgo, 2010). Therefore, a better understanding of the physiological processes that either precede or accompany the development and progression of PDAC should be a major priority. Mounting evidence suggests that PDAC is highly associated with diabetes, as exemplified by the fact that almost 80% of patients diagnosed with PDAC are simultaneously diagnosed with diabetes (McAuliffe & Christein, 2013; Tan et al, 2017). Also, diabetic patients have 50% more risk of developing PDAC than healthy individuals, raising the intriguing question as to whether diabetes would facilitate PDAC formation, or, instead, diabetes occurs as a consequence of PDAC development (McAuliffe & Christein, 2013; Tan et al, 2017). Yet, both the mechanisms underlying this intricate association between PDAC and diabetes as well as its clinical relevance remain poorly understood. In the present study, we used a variety of in vivo experimental approaches to interrogate the influence of PDAC and its very earliest preneoplastic lesions PanINs on the integrity and homeostasis of pancreatic islet cells, which govern whole-body glucose availability and utilization.

The *KC* mouse model used in this study relies on the pancreas-specific expression of oncogenic Kras mutant (*Kras*^*G12D*^), which affects more than 90% of PDAC patients (Almoguera et al, 1988; Hezel et al, 2006). In these mice, expression of Kras^G12D^ leads to the formation of early PanIN lesions, which eventually later evolve into late PanINs and PDAC (Hingorani et al, 2003; Tuveson et al, 2004), thus providing us with an outstanding platform for investigating possible alterations in islet endocrine cells during different stages of tumor progression. Strikingly, we found that *KC* mice exhibited massive depletion of β-cells, occurring at any stages of tumorigenesis examined, including very early PanIN lesions. In contrast, all other islet endocrine cells (e.g., α, δ, and PP) appeared to be unaffected by PanINs or even full-blown PDAC. Intriguingly, these endocrine cells retained their normal distribution at the periphery of islets that are void of β-cells, giving rise to hollow structures that we termed remnant islets. Such finding is likely to be clinically relevant, as we were also able to detect remnant islets in human PDAC biospecimens. Based on these findings, we suggest that PDAC initiation might lead to selective depletion of β-cells, which could ultimately result in decreased insulin secretion and attendant hyperglycemia and diabetes. This physiopathological scenario could further create an imbalance in the ratio between β-cells and other endocrine cells insensitive to PDAC, and thus could exacerbate the diabetes phenotype (see model in Fig 6C). Of relevance, recent studies have shown that most of PDAC patients diagnosed with diabetes experience a significant increase in systemic glucagon/insulin ratio (Kolb et al, 2009), a phenomenon postulated to facilitate gluconeogenesis and glucose release to the circulatory system, as glucagon is known to oppose insulin’s ability to suppress glucose production by the liver. Nonetheless, how and when this phenomenon takes place during the course of tumorigenesis has remained unexplored. Therefore, our findings that early PanINs can trigger depletion of β-cells without affecting α-cells not only provide a mechanistic explanation for the increased glucagon/insulin ratio in PDAC patients but also reveal that this alteration might occur very early during PDAC pathogenesis. This raises the tantalizing possibility that the insulin/glucagon ratio may be used as a marker for the emergence of PDAC, for which no effective diagnostic method is currently available (Hidalgo, 2010).

In addition to glucagon, our study also reveals that PanINs and/or PDAC do not affect other islet cell types, including δ- and PP cells. δ-cells produce somatostatin, which has been shown to inhibit insulin secretion, suggesting that an imbalance between β- and δ-cells might also contribute to the diabetes complication associated with PDAC (Hauge-Evans et al, 2009; Jain & Lammert, 2009). For PP, there has not been any decisive consensus as to whether this hormone contributes to energy homeostasis and diabetes by directly regulating insulin production, action, or both (Yulyaningsih et al, 2014). Therefore, future investigations will be required to establish whether an imbalance between β- and PP cells plays a causative role in diabetes development under PDAC circumstances.

Besides PDAC, diabetes is a comorbidity associated with other severe conditions, such as obesity and pancreatitis. Obesity induces diabetes by a combination of β-cell failure and insulin resistance in peripheral tissues, whereas pancreatitis causes β-cell distress due to excessive inflammation that affects the whole pancreatic parenchyma (Donath et al, 2013; Ewald & Hardt, 2013; Kleeff et al, 2017; Malhi & Camilleri, 2017). In addition, both obesity and diabetes represent major risk factors for PDAC (Guerra et al, 2011; Malhi & Camilleri, 2017). In efforts to delineate whether PDAC shares similar mechanisms with obesity and pancreatitis that lead to the deregulation of glucose homeostasis, we analyzed islets from mice subjected to long-term high-fat diet feeding until the development of obesity or islets from mice injected with cerulean, which provokes pancreatic alterations with features similar to those seen in human pancreatitis. Although both mouse models of obesity and pancreatitis developed overt diabetes, we were not able to see any significant depletion of β-cells or formation of remnant islets, suggesting that PDAC might promote diabetes by mechanisms distinct from obesity and pancreatitis. At present, whether PDAC development also culminates in β-cell failure, insulin resistance, or β-cell distress (similar to what occurs in obesity and pancreatitis, respectively) in addition to driving erosion of β-cell mass requires further investigation.

Probing mechanisms of PDAC-mediated islet β-cell depletion, we detected a significant increase in the abundance of cleaved caspase 3, a prominent hallmark of apoptotic cell death (Kurokawa & Kornbluth, 2009). Based on the literature (Friess et al, 1993; Bardeesy et al, 2006; Zhao et al, 2012; Dhawan et al, 2016), we speculate that tumor-derived factors produced from the extensive desmoplastic reactive stroma associated with PDAC might trigger apoptosis of β-cells. We focused our attention on TGF-β, whose production is known to rise sharply during PDAC development and progression (Friess et al, 1993; Bardeesy et al, 2006). Paradoxically, although loss of Smad4 or TβRII has been noted in many PDAC patients and postulated to cause loss of TGF-β cytostatic signaling (Hezel et al, 2006; Iacobuzio-Donahue, 2012; Cancer Genome Atlas Research Network, 2017), increased TGF-β production from both PDAC cells and their supporting stroma is predictive of aggressive clinicopathological characteristics, being associated with poor prognosis and high mortality in PDAC patients (Friess et al, 1993). Given its prominent role in the epithelial-to-mesenchymal transition (EMT) process and cell migration at late stages of PDAC (Massague, 2008; David et al, 2016), a model that arises from previous studies is that TGF-β might exacerbate PDAC progression by fostering the invasive behaviors of cancer cells, thereby leading to widespread metastasis and general organ dysfunction. However, to the best of our knowledge, there has not been any direct in vivo evidence that TGF-β signaling indeed aggravates the outcome of PDAC exclusively owing to its ability to regulate EMT and associated cell invasion and metastasis. In agreement with the general notion (Friess et al, 1993; Bardeesy et al, 2006), we detected a marked increase in TGF-β signaling in PDAC tissues both within and outside the islets, as assessed by the phosphorylation of Smad2 and accumulation of the TGF-β target genes *JunB*. It is also worth mentioning that the increase in TGF-β signaling occurs even at the very early preneoplastic lesions, suggesting that TGF-β might fulfill an activity distinct from its postulated pro-metastatic role that typically manifests at late stages of PDAC. In this study, we found that activation of TGF-β signaling was associated with increased caspase 3 cleavage within islets, suggesting that activation of TGF-β signaling might cause apoptotic β-cell death during PDAC progression. In support of this hypothesis, systemic elevation of TGF-β levels in healthy animals was sufficient to induce apoptotic β-cell death. Moreover, genetic inactivation of the TGF-β pathway through ablation of either *Smad4* or *TβRII* almost completely blocked apoptotic β-cell death and remnant islet formation, which is consistent with the abundant literature that TGF-β induces apoptotic cell death through a mechanism involving at least canonical Smad signaling. A similar conclusion could be reached from immunopharmacological experiments using a pan-neutralizing TGF-β antibody, highlighting TGF-β as an attractive drug candidate for the treatment of diabetes that may accompany PDAC. From these studies, it is becoming clear that elaboration of potent strategies to inactivate TGF-β should be an important therapeutic goal to restore the insulin levels needed to maintain normal activity and good quality of life and ultimately prolong the lives of PDAC patients. However, given the paradoxical roles of TGF-β signaling pathway as a tumor suppressor and tumor promoter during cancer progression, a comprehensive understanding of how TGF-β signaling functions in PDAC using inducible genetic models and integrative proteomic and single-cell sequencing approaches is a prerequisite for ascertaining whether antagonizing this pathway is prudent for diabetic therapeutics when its tumor-suppressive features might co-exist. Such comprehensive studies could also lead to the identification of components of the TGF-β signaling pathway that operate exclusively in β-cells, which could explain why TGF-β signaling drives apoptosis selectively in β-cells.

Overall, the molecular framework that we propose will contribute to the elucidation of mechanistic paradigms of diabetes during PDAC progression, and may ultimately lead to the development of effective strategies to curb PDAC-driven diabetes, or even to the elaboration of innovative methods for early diagnosis of PDAC, which usually manifests without early symptoms, rendering detection of PDAC quite challenging.

## Materials and Methods

### Reagents

The primary antibodies used in this study were Cleaved Caspase 3 (9661; Cell Signaling Technology), Cytokeratin 19 (ab52625; Abcam), Glucagon (8233; Cell Signaling Technology), Glucagon (G2654; Sigma-Aldrich), Insulin (8138; Cell Signaling Technology), CD31 (77699s; Cell Signaling Technology), Insulin (3630; Cell Signaling Technology), JunB (3753; Cell Signaling Technology), Mucin5AC (MA5-12178; Thermo Fisher Scientific), PP (ab77192; Abcam), Phospho-Smad2 (3101; Cell Signaling Technology), Phospho-Smad2 (8828; Cell Signaling Technology), Smad2 (5339; Cell Signaling Technology), Somatostatin (MA516987; Thermo Fisher Scientific), and Sox9 (ABE571; Millipore). For immunoneutralization, anti–TGF-β and its matching-isotype IgG were obtained from BioXcell (BE0057 and BE0089, respectively). For immunofluorescent staining, the secondary antibodies used were Donkey antigoat Alexa-Fluor 448 (A11055; Life Technologies), Goat antimouse Alexa-Fluor 568 (A11004; Invitrogen), Goat antirabbit Alexa-Fluor 568 (A11011; Invitrogen), and Goat antirat Alexa-Fluor 568 (A11077; Life Technologies). For immunohistochemical staining, the secondary antibodies used were biotin-conjugated secondary antibodies in specific kits obtained from Vector Laboratories (mouse kit, PK-6102; rabbit kit, PK-6101). DAB peroxidase substrate was obtained from Vector Laboratories (SK-4100). High-fat diet was obtained from Envigo (TD.06414). Caerulein (C9026) and tamoxifen (T5648) were obtained from Sigma-Aldrich. D-luciferin was obtained from PerkinElmer (122799). TGF-β1 was obtained from Sigma-Aldrich (T7039).

### Mice treatment and analysis

All animal experiments were approved by the Institutional Animal Care and Use Committee of the University of Mississippi Medical Center.

*Loxp-Stop-Loxp-Kras.G12D* (*LSL.Kras*^*G12D*^), *Loxp-Stop-Loxp-Luc*, and *Pdx1-Cre* were obtained from the National Cancer Institute Mouse Repository. *Smad4*^*fl/fl*^, *Ins2-Cre*^*ERT2*^, *mTmG (Gt(ROSA)26Sor*^*tm4(ACTB-tdTomato,-EGFP)Luo*^*/J)*, and *TβRII* ^*fl/fl*^ mice were obtained from The Jackson Laboratory. The mouse models used in this study were generated through successive crossbreeding as appropriate. Their genotypes are as follows:

-*KC: LSL-Kras*^*G12D*^*;Pdx1-Cre*
-*KSC: LSL-Kras*^*G12D*^*;Smad4*^*fl/fl*^*;Pdx1-Cre*
-*KTC: LSL-Kras*^*G12D*^*;TβRII* ^*fl/fl*^*;Pdx1-Cre*
-*mTmG: mTmG;Pdx1-Cre*
-*KC-mTmG: LSL-Kras*^*G12D*^*;mTmG;Pdx1-Cre*
-*β-Luc*^*Tam*^*: LSL-Luc;Ins2-Cre*^*ERT2*^
-*KβLuc*^*Tam*^*: LSL-Kras*^*G12D*^*;LSL-Luc;Ins2-Cre*^*ERT2*^

All mice were maintained on a mixed C57BL/6J and FVB/N genetic background. Mice were maintained in 12-h light:dark cycles (6:00 am–6:00 pm) at 22°C and fed a standard rodent chow diet. Most of the experiments involve 6-mo-old mice, age at which the vast majority of *KC* mice develop PanINs, and only a small fraction of *KC* mice (about 15%) also contains small areas of full-blown PDAC. All mice were included in this study irrespective of their PanIN or PDAC contents, gender, health, or genetic background. For the study involving obesity-induced diabetes, siblings of *KC* mice (without Cre) were fed with food containing 60% fat for 24 wk and provided with water ad libitum. For pancreatitis studies, siblings of *KC* mice (without Cre) were injected with caerulein (50 μg/kg) or vehicle (saline) on two alternating days once every hour for 6 h each day. *KC* mice used in obesity and pancreatitis experiments were backcrossed into the C57BL/6J background for three generations. For studies involving inducible *Ins2Cre*^*ERT2*^, tamoxifen was dissolved in sterile corn oil and administered subcutaneously at 100 mg/kg per injection for five consecutive days. D-luciferin was dissolved in PBS and administered by intraperitoneal injection at 75 mg/kg body weight before imaging. Control mice received solvent only. For in vivo imaging, the mice were anaesthetized by isoflurane inhalation and imaged after injection of D-luciferin using a Xenogen IVIS Spectrum (Caliper Life Sciences). For anti–TGF-β studies, the mice were divided into different treatment groups randomly while satisfying the criteria that the average body weight in each group would be about the same. For the neutralization experiment, anti–TGF-β or matching-isotype IgG was injected intraperitoneal at 100 mg/kg body weight twice weekly for 4 mo. For the TGF-β treatment experiment, TGF-β1 was reconstituted in saline solution and injected intraperitoneally at 0.7 mg/kg body weight once daily for three consecutive days.

### Immunoblotting

Cell extracts were prepared in lysis buffer containing 50 mM Tris HCl (pH = 8.0), 120 mM NaCl, 5 mM EDTA, 1% IGEPAL, protease inhibitors (5872; Cell Signaling Technology), and phosphatase inhibitors (Calbiochem). Protein concentrations were determined by using the Pierce BCA Protein Assay Kit (#23225; Thermo Fisher Scientific) and the samples were denatured using SDS sample buffer (#1610747; Bio-Rad). The samples were loaded into a Criterion TGX Any kD gel (#5671124; Bio-Rad) and separated by electrophoreses at constant milliamperes at 40 mA. The gels were then transferred onto a nitrocellulose membrane (#1620115; Bio-Rad) by a wet transfer system (Bio-Rad) and stained with Ponceau S Solution (P7170-1L; Sigma-Aldrich) and blocked by incubation with 5% dry milk in TBST (TBS with 0.2% Tween20). The membranes were probed with the anti–TGF-β (1:1,000) in blocking buffer at 4°C, washed with TBST, and incubated with the peroxidase-conjugated secondary antibody. ECL Western blotting substrates (RPN2236; GE Healthcare) were used for visualization of the results.

### Measurements of blood glucose and insulin levels and glucose tolerance

To measure blood glucose, the mice underwent a morning fast (5–6 h). A blood sample was collected from an unrestrained mouse by cutting off the tip of its tail and measured using ReliOn Prime glucose monitor system. A minimum of two blood measurements were taken per mice. To perform a glucose tolerance test, the mice were fasted for 5–6 h, and blood glucose measurements were obtained for baseline comparison. Subsequently, the mice were injected with 20% glucose (2 g/kg) intraperitoneally, and blood glucose was measured at 30-min intervals for a span of 2 h. To measure plasma insulin levels, blood was collected and centrifuged at 11,180*g* for 15 min to collect the serum. Insulin levels were obtained by ELISA according to the manufacturer’s method (Alpco Insulin Mouse, 80-INSMS-E01). Briefly, plasma was added to microplates coated with monoclonal antibody specific for insulin with a conjugate and incubated at room temperature on a shaker at a horizontal movement speed of 700 per min for 2 h. Subsequently, the wells were washed and the substrate was added to each well and incubated at room temperature for a second time. Finally, a stop solution was added and the optical density was measured by a spectrophotometer (SpectraMax M3) at 450 nm.

### IF

Tissue samples were fixed in 10% formalin and embedded in paraffin. Human tissue microarrays were obtained from Biomax (PA482). Tissue sections were deparaffinized with xylene and rehydrated in a graded series of ethanol. Antigen retrieval was performed for 30 min at high temperature in citrate buffer. For frozen section preparation, the tissues were collected and immersed in 4% paraformaldehyde followed by immersion in 10% sucrose solution and then mounted in OCT embedding compound. Then, the slides were blocked and incubated overnight with the appropriate antibody at 4°C. Finally, the slides were incubated with the secondary antibodies conjugated to Alexa-Fluor 568 or Alexa-Fluor 448 and co-stained with DAPI. The slides were viewed on a fluorescence microscope (Nikon Eclipse 80*i*), and the images were captured with Nis Elements BR 3.2 64 bit. In both IHC and IF experiments, the islet areas were delineated by immunostaining using antibodies to insulin (center) or glucagon, PP or somatostatin (periphery). The islets were classified as normal, irregular (distorted shape), or remnant (depleted of β-cells). To quantify islet cells, all islets within six tissue sections (10–22 islets per section) were included, and at least six mice per group were analyzed. The numbers of β-cells in islets vary between 6 and 97, depending on the genotype and age. A full description of the mouse models used is provided in Table 1.

**Table 1.** Description of mouse models of pancreatic ductal adenocarcinoma (PDAC) used, including genotype, number of mice in each group, onset of PanINs (or intraductal papillary mucinous neoplasia [IPMN] for *KSC*) and PDAC, and percentage of remnant islets in mice with PanINs only or both PanINs and PDAC. None: no remnant islets, PanIN or PDAC lesions were found. N/P: mice did not show PanIN only. T/A: PDAC was analyzed when the mice were euthanized.

Mice genotype	Onset PanIN only (mo)	Onset PDAC + PanIN (mo)	% Remnant islet (PanIN only)	% Remnant islet (PanIN + PDAC)
KC (n = 38)	4–6	5–12	26% (5 mice)	48% (19 mice)
*KC*+*anti–TGF*-β (n = 6)	6 (T/A)	6 (T/A)	0% (2 mice)	6% (4 mice)
*KC−mTmG* (n = 6)	None	None	None	None
*KSC* (n = 6)	1–4 (IPMN)	4–8	N/P	3% (6 mice)
*Smad4*^*KO*^ (n = 3)	None	None	None	None
*KTC* (n = 6)	1–2	1–2	N/P	0% (6 mice)
*mTmG* (n = 6)	None	None	None	None
β-*Luc*^*Tam*^ (n = 6)	None	None	None	None
*KβLuc*^*Tam*^ (n = 6)	None	None	None	None
*HFD* (n = 6)	None	None	None	None
*Pancreatitis* (n = 6)	None	None	None	None

### Pancreas histology

Tissue samples were fixed in 10% formalin and embedded in paraffin, and the sections were deparaffinized with xylene and rehydrated in a graded series of ethanol. The slides were then stained with hematoxylin and washed with water. After counterstaining with eosin, the slides were dehydrated in alcohol and cleared in xylene.

For IHC, pancreatic FFPE sections were immunostained with antibodies to cytokeratin 19 (CK19, 1:100), pSmad2 (1:100), anti-Muc5Ac (1:100), Sox9 (1:100), insulin (1:100), CD31 (1:100), or JunB (1:100). The slides were then washed, incubated with the appropriate secondary antibody conjugated with peroxidase, and revealed by DAB using standard techniques.

For both pancreas histology and IHC, the slides were mounted using Permount (SP15-500; Thermo Fisher Scientific), viewed on a bright-field microscope (Nikon Eclipse 80*i*), and the images were captured with Nis Elements BR 3.2 64 bit.

### Statistical analysis

Statistical significance between two groups was evaluated using the unpaired *t* test, assuming a two-tailed distribution. Multiple comparisons between three or more groups were performed using one-way or two-way ANOVA; *P < 0.05; **P < 0.01; ***P < 0.001; ns, nonsignificant. Data are presented as mean ± SEM or as dot plot with a line at the median and whiskers showing SD. The error bars (SEM) were derived from at least three independent biological replicates, not technical.

## Supplementary Material

Reviewer comments
